# Implicit Detection Observation in Different Features, Exposure Duration, and Delay During Change Blindness

**DOI:** 10.3389/fpsyg.2020.607863

**Published:** 2021-01-08

**Authors:** Wang Xiang

**Affiliations:** ^1^Faculty of Psychology, Tianjin Normal University, Tianjin, China; ^2^School of Education Science, Guangxi University for Nationalities, Nanning, China

**Keywords:** Implicit detection, change blindness, stimulus feature, exposure duration, delay, set size

## Abstract

To investigate whether implicit detection occurs uniformly during change blindness with single or combination feature stimuli, and whether implicit detection is affected by exposure duration and delay, two one-shot change detection experiments are designed. The implicit detection effect is measured by comparing the reaction times (RTs) of baseline trials, in which stimulus exhibits no change and participants report “same,” and change blindness trials, in which the stimulus exhibits a change but participants report “same.” If the RTs of blindness trials are longer than those of baseline trials, implicit detection has occurred. The strength of the implicit detection effect was measured by the difference in RTs between the baseline and change blindness trials, where the larger the difference, the stronger the implicit detection effect. In both Experiments 1 and 2, the results showed that the RTs of change blindness trials were significantly longer than those of baseline trials. Whether under set size 4, 6, or 8, the RTs of the change blindness trials were significantly longer than those in the baseline trials. In Experiment 1, the difference between the baseline trials’ RTs and change blindness trials’ RTs of the single features was significantly larger than that of the combination features. However, in Experiment 2, the difference between the baseline trials’ RTs and the change blindness trials’ RTs of single features was significantly smaller than that of the combination features. In Experiment 1a, when the exposure duration was shorter, the difference between the baseline and change blindness trials’ RTs was smaller. In Experiment 2, when the delay was longer, the difference between the two trials’ RTs was larger. These results suggest that regardless of whether the change occurs in a single or a combination of features and whether there is a long exposure duration or delay, implicit detection occurs uniformly during the change blindness period. Moreover, longer exposure durations and delays strengthen the implicit detection effect. Set sizes had no significant impact on implicit detection.

## Introduction

Change blindness refers to an inability of an observer to detect a change in a visual scene even if they have good eyesight and know that a change is imminent (Simons and Rensink, 2005; Rensink, 2018). Studies have shown that although changed stimuli cannot be reported consciously, it is partially processed unconsciously (Beck and van Lamsweerde, 2011; Ball et al., 2015; Chetverikov et al., 2018).

In a two-alternative forced-choice task, the accuracy of guessing the object that changes its orientation (Fernandez-Duque and Thornton, 2000; Fernandez-Duque et al., 2003; Laloyaux et al., 2006) or position (Smilek et al., 2000; Piasecki et al., 2017) is above the chance level, and the reaction times (RTs) of change blindness trials is longer than that of no-change trials (Fernandez-Duque and Thornton, 2003; Koivisto and Revonsuo, 2003). Studies using eye-tracking showed that the gaze duration at the change location was significantly longer than at other locations (Hollingworth et al., 2001; Delvenne et al., 2007). These results indicate that, to a certain extent, the change has been registered and located (Reynolds and Withers, 2015). Electrophysiological results (Busch, 2013; Lyyra et al., 2014; Ball et al., 2015; Hadid and Lepore, 2017; Scrivener et al., 2019), brain imaging (Kiat et al., 2018), and brain stimulation (Morgan et al., 2013; Hsu et al., 2014; Lyyra, 2014) also support implicit detection consistently. Although the existence of implicit detection has been verified, little is known about the influencing factors, which necessitates further investigation.

The Feature-Integration Theory proposes that some visual features are processed in parallel in a “pre-attentive” front end (Treisman and Gelade, 1980). The visual features of objects can be integrated only within the focal attention range (Treisman and Gelade, 1980; Treisman, 1998). Static display visual search experiments have demonstrated that only orientation is pre-attentive (Yu and Lisin, 2013), whereas complex orientation cannot be processed without awareness when processing other features (Rajimehr, 2004). It is not clear whether implicit detection occurs when exposed to a single feature, or a combination feature such as Gabor patches. We aim to investigate this in the current study.

Individuals require at least 400 ms to process and consolidate images into their visual working memory (Potter, 1976). Therefore, prolonging the exposure duration of a stimulus may increase the rate of change detection. Rensink et al. (1997) found that when the exposure duration of a stimulus was extended from 240 to 560 ms, a subject’s ability to detect change did not improve. Whether implicit detection effects are stronger with longer exposure duration is an issue to be investigated.

Studies have indicated that even when consciously unaware of changes, observers still retained information about the pre- and post-change objects (Mitroff et al., 2004). However, this information lasts only half a second before being replaced by new stimuli (Simons and Rensink, 2005). Using the forced-choice task, it was found that when the delay between new and old stimuli exceeded 70 ms, the subject’s ability to detect changes decreased (Phillips, 1974). Whether the implicit detection effect is weaker with longer delays is another issue that requires exploration.

The purpose of the current study was to explore (1) whether implicit detection can occur with a single feature or a combination of features, (2) whether the implicit detection effect is stronger with longer exposure duration, and (3) whether the implicit detection effect is weaker with longer delays.

We measured the implicit detection effect by comparing the RTs of the baseline trials, in which the stimulus exhibits no change and participants report “same,” and the change blindness trials, in which the stimulus exhibits a change but participants report “same.” This measure provides a method to assess implicit or covert change detections (Williams and Simons, 2000; Koivisto and Revonsuo, 2003; Lin and Murray, 2014). If the RTs are significantly longer for when a change is not detected than when there is no change, it indicates that implicit detection has occurred (Mitroff et al., 2002). The strength of the implicit detection effect was measured by the difference in RTs between the baseline and change blindness trials, where the larger the difference, the stronger the implicit detection effect.

## Materials and Methods

### Participants

Thirty college students (16 men and 14 women; mean age 19.3 years; age range 18–23 years) volunteered to take part in Experiment 1. Another 30 college students (13 men and 17 women; mean age 18.8 years; age range 17–22 years) volunteered to take part in Experiment 2. All participants had normal or corrected-to-normal visual acuity and had no self-reported color blindness.

### Stimuli and Apparatus

**FIGURE 1:**
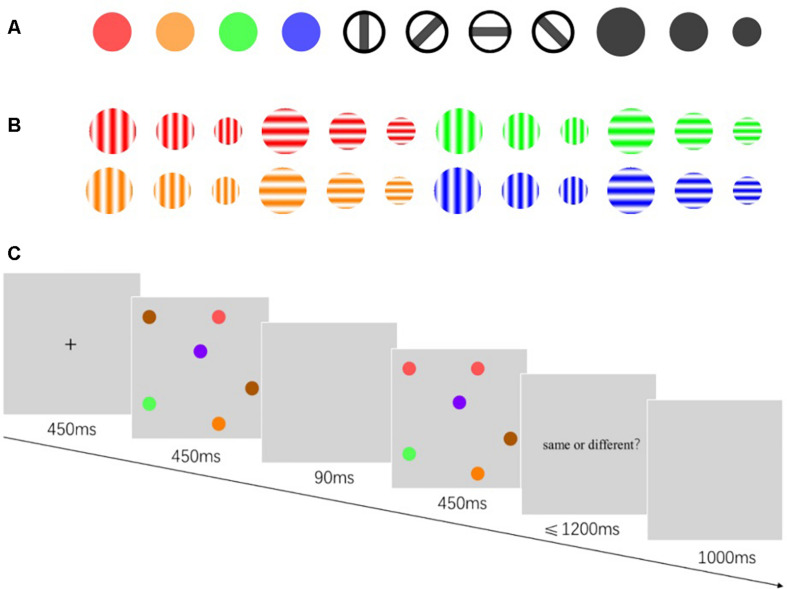
Illustration of stimulus and the progression of a trial. **(A)** Illustration of stimuli used in Experiments 1a and 2a. The colour name and RGB values were as follows: red (255,0,0), orange (255,128,0), green (0,255,0), blue (0,0,255). **(B)** Illustration of stimuli used in Experiments 1b and 2b. The Gabor patches were produced by an online Gabor-patch generator (https://www.cogsci.nl/pages/gabor-generator), and the parameter values were as follows: Orientation, 0°/90°; envelope, circular (sharp edge); frequency, 0.04; phase, 0. **(C)** Illustration of the progression of a trial for Experiment 1a. The exposure duration and delay was different in Experiments 1 and 2.

Experiments 1 and 2 were composed of two sub-experiments: “a” and “b.” In Experiments 1a and 2a, there were three types of stimuli (Figure 1A): color, size, and orientation. The color stimulus was disks of different colors with a visual angle diameter of 1°. The size stimulus was a black disk with three sizes: large, medium, and small, and the diameters were at visual angles of 1.25°, 1°, and 0.75°, respectively. The orientation stimulus was a circle containing a black rectangle. The circle diameter was at a 1° visual angle. The black rectangle was at a 1° × 0.2° visual angle, and had four orientations, vertically inclined at 0°, 45°, 90°, and 135°.

In Experiments 1b and 2b, the stimulus was Gabor patches with different colors, sizes, and orientations (Figure 1B). The color, size, and orientation parameters of the Gabor patches corresponded to those in Experiments 1a and 2a. The stimulation display was composed of different numbers of stimulation randomly distributed in an imaginary 4 × 4 grid. The grid was at a visual angle of 10° × 10° wide. The experimental program was written in PsychoPy 3.0 (Peirce et al., 2019). The screen resolution was 1,024 × 768 pixels, with a refresh rate of 60 Hz.

### Design and Procedure

The one-shot change detection paradigm was adopted. This paradigm requires a participant to remember as much of the pre-change display as possible, to increase the odds of detecting the target (Jensen et al., 2011). The task is often used to test implicit detection (Kimura et al., 2008; Caudek and Domini, 2013; Nishiyama and Kawaguchi, 2014).

Each sub-experiment was then divided into three blocks. In Experiments 1a and 1b, each block differs only in the exposure duration of the stimulation display. The exposure duration was 250, 450, or 900 ms. Each block had three set sizes: 4, 6, and 8.

The participants sat with their eyes 60 cm away from the center of the screen. After the instruction is presented (Figure 1C), the fixation point “+” was displayed in the screen center for 450 ms. Subsequently, display A was presented for 250, 450, and 900 ms. After a blank screen showed for 90 ms, display A′ was shown for 250, 450, and 900 ms. Displays A and A′ were either identical or different. In the case where they were different in Experiment 1a, a single object in display A′ has changed its color, size, or orientation. In Experiment 1b, one of the objects in display A′ changed two of its three features (i.e., color, size, and orientation) simultaneously. After display A′ disappeared, the reaction prompt was presented. Participants were required to report whether displays A and A′ were the same within 1,200 ms by pressing a corresponding key. After pressing the key, the screen immediately went blank for 1,000 ms, and the next trial was performed.

Based on previous experiments (Beck et al., 2001; Fernandez-Duque and Thornton, 2003; Laloyaux et al., 2006), we set one third of trials as no-change trials. Each block was composed of 270 trials, including 90 no-change trials and 180 change trials. The trials of each set size and change type were equal.

Experiments 2a and 2b were similar to Experiments 1a and 1b, except that the exposure duration was 450 ms, and the delay blank was 120, 250, and 450 ms.

### Data Analysis

Using stimulus change as the signal, we calculated the false alarm rate (FA) and hit rate (H) based on the signal detection theory. Participants whose FA ≥ H were excluded, as it meant that the participants did not understand the requirements or did not cooperate (Laloyaux et al., 2006). We used d_L_ as a discrimination sensitivity index, as it has the fewest calculation errors (Thornton and Fernandez-Duque, 2000).

$$d_{L}=\ln\left\{\left[\text{H}\,\left(1-\text{FA}\right)\right]/\left[\left(1-\text{H}\right)\,\text{FA}\right]\right\}$$

The RTs were entered into an analysis of variance (ANOVA) with three within-subject variables: trial types, exposure/delay time, and set sizes. The Bonferroni method was used for multiple and *post hoc* comparisons.

## Results

The average RTs of baseline and blindness trials under different exposure durations in Experiment 1 are provided in Table 1. The average RTs of baseline and blindness trials under different delay times in Experiment 2 are provided in Table 2.

**TABLE 1:** The average reaction time (in ms) in Experiments 1a and 1b.

		Set size 4	Set size 6	Set size 8	Total
**Experiment 1a**					
250 ms	Baseline	434.21 (96.90)	408.78 (76.22)	368.46 (91.26)	403.81 (67.65)
	Blindness	505.18 (114.13)	436.57 (73.70)	398.15 (81.06)	446.63 (68.29)
450 ms	Baseline	289.13 (128.61)	289.59 (101.16)	289.53 (115.21)	289.42 (91.93)
	Blindness	370.73 (130.68)	406.46 (129.25)	372.45 (120.43)	383.22 (101.27)
900 ms	Baseline	384.92 (120.18)	356.21 (123.59)	323.45 (133.33)	403.81 (67.65)
	Blindness	532.32 (137.48)	418.52 (125.80)	418.63 (112.33)	446.63 (68.29)
**Experiment 1b**					
250 ms	Baseline	414.97 (125.68)	357.49 (85.05)	354.77 (106.32)	375.74 (90.91)
	Blindness	478.88 (113.06)	422.91 (121.92)	395.10 (112.27)	432.30 (93.88)
450 ms	Baseline	295.43 (124.11)	274.77 (109.22)	268.14 (105.67)	279.44 (85.63)
	Blindness	356.95 (149.93)	328.14 (107.58)	325.46 (90.37)	336.85 (93.08)
900 ms	Baseline	402.13 (154.23)	373.48 (116.61)	376.34 (129.10)	383.99 (106.58)
	Blindness	386.88 (134.52)	446.35 (167.95)	420.02 (126.10)	412.67 (115.58)

*Standard deviations appear in parentheses.*

**TABLE 2:** The average reaction time (in ms) in Experiments 2a and 2b.

		Set size 4	Set size 6	Set size 8	Total
**Experiment 2a**					
120 ms	Baseline	307.23 (126.95)	298.97 (125.53)	323.68 (127.17)	309.96 (107.27)
	Blindness	392.76 (128.96)	345.35 (133.78)	346.00 (114.13)	361.37 (105.20)
250 ms	Baseline	302.82 (121.46)	330.13 (127.56)	324.56 (122.09)	319.17 (99.93)
	Blindness	383.60 (131.99)	403.00 (145.36)	375.75 (118.98)	387.45 (107.85)
450 ms	Baseline	419.28 (138.23)	438.21 (119.89)	393.89 (138.89)	417.13 (102.67)
	Blindness	525.39 (124.23)	492.68 (133.66)	488.04 (143.42)	502.04 (99.82)
**Experiment 2b**					
120 ms	Baseline	353.58 (139.00)	340.29 (121.19)	344.24 (117.37)	346.04 (109.36)
	Blindness	499.81 (117.55)	429.92 (146.13)	413.76 (124.15)	447.83 (98.09)
250 ms	Baseline	339.06 (129.50)	252.16 (103.60)	288.25 (81.39)	293.16 (80.28)
	Blindness	463.56 (144.49)	400.99 (93.19)	417.39 (97.35)	427.31 (73.65)
450 ms	Baseline	394.95 (141.32)	370.04 (106.67)	392.98 (93.25)	385.99 (72.53)
	Blindness	560.16 (153.32)	527.69 (134.79)	567.86 (122.62)	551.90 (95.21)

*Standard deviations appear in parentheses.*

### Results of Experiment 1a

Participants’ average H was 61.05%. The Spearman’s correlation coefficient between exposure duration and H was 0.30, *p* = 0.005. The one-sample *t* test showed that *d*_*L*_ was significantly greater than 0. The ANOVA showed that the RTs of baseline trials were significantly shorter than that of blindness trials, *F*(1, 522) = 66.14, *p* < 0.001, and η*_*p*_*^2^ = 0.11. The RTs between 250, 450, and 900 ms exposure durations were significantly different, *F*(2, 522) = 30.52, *p* < 0.001, and η*_*p*_*^2^ = 0.11. Multiple comparisons showed that the RTs for an exposure duration of 250 ms was significantly longer than the RTs for an exposure duration of 450 ms (*p* < 0.001). The RTs for 450 ms exposure duration were significantly shorter than that of 900 ms exposure duration (*p* < 0.001). The RTs among those of set size 4, 6, and 8 were significantly different, *F*(2, 522) = 11.71, *p* < 0.001, and η*_*p*_*^2^ = 0.04. Multiple comparisons showed that the RTs of set size 4 were significantly longer than that of set size 6 (*p* = 0.016) and 8 (*p* < 0.001).

The interaction effect of trial type × exposure duration was significant, *F*(2, 522) = 3.56, *p* = 0.029, η*_*p*_*^2^ = 0.01. *Post hoc* comparisons showed that the RTs of baseline trials were significantly shorter than those of change blindness trials when exposure durations were 250 (*p* = 0.012), 450 (*p* < 0.001), and 900 ms (*p* < 0.001). There was no significant interaction effect of trial type × set size, *F*(2, 522) = 1.11, *p* = 0.330, η*_*p*_*^2^ = 0.01. The interaction effect of exposure duration × set size was significant, *F*(4, 522) = 3.80, *p* = 0.005, η*_*p*_*^2^ = 0.03. *Post hoc* comparisons showed that the RTs of set size 4 were significantly longer than that of set size 8 (*p* < 0.001) when exposure duration was 250 ms, whereas the RTs of set size 4 was significantly longer than that of set size 6 (*p* = 0.002) and 8 (*p* < 0.001) when exposure duration was 900 ms. There was no significant interaction effect of trial type × exposure duration × set size, *F*(4, 522) = 1.10, *p* = 0.357, and η*_*p*_*^2^ = 0.01.

**FIGURE 2:**
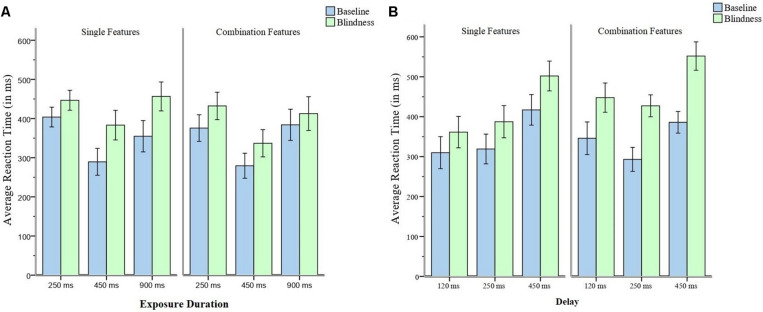
**(A)** The Average RTs of baseline trials and that of blindness trials in Experiment 1. **(B)** The Average RTs of baseline and blindness trials in Experiment 2. Error bars represent 95% confidence intervals.

The RTs of the baseline trials were subtracted from the RTs of the change blindness trials (Figure 2A). The differences among those of 250, 450, and 900 ms exposure was significantly different, *F*(2, 58) = 5.68, *p* = 0.006, and η*${}_{p}^{2}$* = 0.16. Multiple comparisons showed that the difference in the RTs of 250 ms exposure was significantly smaller than that of 450 ms, *p* = 0.006. Similarly, the difference in the RTs of 250 ms exposure was significantly smaller than that of 900 ms, *p* = 0.008.

### Results of Experiment 1b

Participants’ average H was 68.11%. The Spearman’s correlation coefficient between exposure duration and H was 0.06, *p* = 0.586. The one-sample *t* test showed that *d*_*L*_ was significantly greater than 0. The ANOVA showed that the RTs of baseline trials were significantly shorter than that of change blindness trials, *F*(1, 522) = 20.65, *p* < 0.001, and η*_*p*_*^2^ = 0.04. The RTs between exposure durations of 250, 450, and 900 ms were significantly different, *F*(2, 522) = 35.24, *p* < 0.001, and η*_*p*_*^2^ = 0.12. Multiple comparisons showed that RTs for 250 ms exposure duration were significantly longer than RTs for 450 ms exposure duration (*p* < 0.001), and the RTs for 450 ms exposure duration were significantly shorter than that of 900 ms exposure duration (*p* < 0.001). The RTs among those of set sizes 4, 6, and 8 were not significantly different, *F*(2, 522) = 2.83, *p* = 0.06, and η*_*p*_*^2^ = 0.01. There was no significant interaction effect of trial type × exposure duration, *F*(2, 522) = 0.81, *p* = 0.444, and η*_*p*_*^2^ = 0.01. There was no significant interaction effect of trial type × set size, *F*(2, 522) = 0.79, *p* = 0.453, and η*_*p*_*^2^ = 0.01. There was no significant interaction effect of exposure duration × set size, *F*(4, 522) = 2.28, *p* = 0.059, and η*_*p*_*^2^ = 0.02. There was no significant interaction effect of trial type × exposure duration × set size, *F*(4, 522) = 1.16, *p* = 0.330, and η*_*p*_*^2^ = 0.01.

The RTs of baseline trials were subtracted from the RTs of the blindness trials (Figure 2A). The differences among those of 250, 450, and 900 ms exposure was not significantly different, *F*(2, 58) = 1.41, *p* = 0.253, and η*${}_{p}^{2}$* = 0.05.

### Results of Experiment 2a

Participants’ average H was 59.37%. The Spearman’s correlation coefficient between delay time and H was 0.27, *p* = 0.010. The one-sample *t* test result showed that *d*_*L*_ was significantly greater than 0. The ANOVA showed that the RTs of baseline trials were significantly shorter than those of change blindness trials, *F*(1, 522) = 37.56, *p* < 0.001, and η*_*p*_*^2^ = 0.07. The RTs between delays of 120, 250, and 450 ms were significantly different, *F*(2, 522) = 48.39, *p* < 0.001, and η*_*p*_*^2^ = 0.16. Multiple comparisons showed that the RTs for a 120-ms delay were significantly shorter than those of a 450-ms delay (*p* < 0.001), and the RTs for a 250-ms delay were significantly shorter than those of a 450-ms delay (*p* < 0.001). The RTs among set sizes 4, 6, and 8 were not significantly different, *F*(2, 522) = 0.50, *p* = 0.609, and η*_*p*_*^2^ = 0.01.

There was no significant interaction effect of trial type × delay, *F*(2, 522) = 0.76, *p* = 0.470, and η*_*p*_*^2^ = 0.01. There was no significant interaction effect of trial type × set size, *F*(2, 522) = 1.04, *p* = 0.356, and η*_*p*_*^2^ = 0.01. The interaction effect of delay × set size was not significant, *F*(4, 522) = 0.85, *p* = 0.496, and η*_*p*_*^2^ = 0.01. There was no significant interaction effect of trial type × delay × set size, *F*(4, 522) = 0.37, *p* = 0.828, and η*_*p*_*^2^ = 0.01.

The RTs of the baseline trials were subtracted from the RTs of the change blindness trials (Figure 2B). The differences among those of 120, 250, and 450 ms delay were not significant, *F*(2, 58) = 1.59, *p* = 0.212, and η*${}_{p}^{2}$* = 0.05.

### Results of Experiment 2b

Participants’ average H was 63.69%. The Spearman’s correlation coefficient between delay time and H was 0.03, *p* = 0.375. The one-sample *t* test showed that *d*_*L*_ was significantly greater than 0. The ANOVA showed that the RTs of baseline trials were significantly shorter than that of change blindness trials, *F*(1, 522) = 162.53, *p* < 0.001, and η*_*p*_*^2^ = 0.24. The RTs between 120-, 250-, and 450-ms delays were significantly different, *F*(2, 522) = 36.94, *p* < 0.001, and η*_*p*_*^2^ = 0.12. Multiple comparisons showed that RTs for a 120-ms delay were significantly longer than those for a 250-ms delay (*p* = 0.014), and the RTs for a 120-ms delay were significantly shorter than those for a 450-ms delay (*p* < 0.001). Furthermore, the RTs for a 250-ms delay were significantly shorter than those for a 450-ms delay (*p* < 0.001). The RTs among set sizes 4, 6, and 8 were significantly different, *F*(2, 522) = 7.25, *p* = 0.001, and η*_*p*_*^2^ = 0.03. Multiple comparisons showed that the RTs of set size 4 were significantly longer than those of set size 6 (*p* = 0.001) and 8 (*p* = 0.048).

The interaction effect of trial types × delay was significant, *F*(2, 522) = 3.10, *p* = 0.046, and η*_*p*_*^2^ = 0.01. Multiple comparisons showed that the RTs of baseline trials were significantly shorter than those of blindness trials when the delay was 120 (*p* < 0.001), 250 (*p* < 0.001), and 450 ms (*p* < 0.001). There was no significant interaction effect of trial type × set size, *F*(2, 522) = 0.34, *p* = 0.716, and η*_*p*_*^2^ = 0.01. The interaction effect of delay × set size was not significant, *F*(4, 522) = 1.24, *p* = 0.294, and η*_*p*_*^2^ = 0.01. There was no significant interaction effect of trial type × delay × set size, *F*(4, 522) = 0.75, *p* = 0.558, and η*_*p*_*^2^ = 0.01.

The RTs of the baseline trials were subtracted from the RTs of the change blindness trials (Figure 2B). The differences among the delays of 120, 250, and 450 ms were significantly different, *F*(2, 58) = 9.70, *p* < 0.001, and η*${}_{p}^{2}$* = 0.25. Multiple comparisons showed that the difference of the 120-ms delay is significantly smaller than that of the 250-ms delay, *p* = 0.030. The difference of the 120-ms delay is significantly smaller than that of the 450-ms delay, *p* < 0.030. The difference of the 250-ms delay is significantly smaller than that of the 450-ms delay, *p* = 0.030.

## General Discussion

The purpose of the current study was to explore (1) whether implicit detection can occur with a single feature or a combination of features, (2) whether the implicit detection effect is stronger with longer exposure duration, and (3) whether the implicit detection effect is weaker with longer delays.

In all four experiments, the discrimination sensitivity index *d*_*L*_ was significantly greater than 0, which indicated that the subjects could distinguish between change and no change. In all four experiments, whether under set sizes 4, 6, or 8, the RTs of the change blindness trials were significantly longer those of the baseline trials. This measure provided a way of assessing implicit change detection (Williams and Simons, 2000). The results indicated that although the participants could not report the changes consciously, they could detect the change implicitly. Regardless of whether a single or a combination of features changed, the length of exposure duration or delay, and the scope of set size, implicit detection was observed.

In Experiments 1a and 1b, the RT difference of baseline and change blindness trials in a single feature differed significantly between exposure durations. In particular, the RTs from 250 ms exposure was significantly shorter than those of 450 and 900 ms. The RT difference of baseline and blindness trials in a combination of features did not differ significantly between exposure durations. We can conclude that with longer exposure durations, the single feature change demonstrated a stronger implicit detection effect, whereas the combination of features change did not show such a stronger effect. The reason for this result may be explained by the Temporal Integration Model. According to this model, change detection includes a temporal process of implicit integration. Explicit change detection with awareness will occur eventually when the accumulated change signal exceeds a certain threshold (Mitroff and Simons, 2002). The change signal increases accumulatively with longer exposure durations. For the single feature change, the accumulated change signal does not exceed the explicit detection threshold, so when the exposure duration was longer, the RT difference between the baseline and change blindness trials was larger. For the combination of features change, the accumulated change signal either reached the maximum or exceeded the explicit detection threshold, so when the exposure duration was longer, the RTs of the baseline and change blindness trials were not significantly different. The participants’ average H for single and combination of features change supports this speculation.

In Experiments 2a and 2b, the RT difference of baseline and blindness trials with different delays were significantly different between single feature and combination of features change. In particular, longer delays produced larger RT differences. In other words, longer delays induce stronger implicit detection effects. We believe that the consolidation process of visual working memory plays an important role. Although attention on related objects may enable perceptual details to enter the visual working memory system, this information can only become a stable representation if it is changed into visual working memory. This transformation process differs from the sensory encoding and maintenance processes. It is a cognitive processing operation that occurs after encoding and before maintenance called consolidation (Woodman and Vogel, 2005; Vogel et al., 2006). In the current study, the delay stage corresponds to the consolidation process. Potter (1976) determined that an individual’s sensory representation could be confused by subsequent information during the transformation into visual working memory, where the longer the delay, the smaller the impact of subsequent information on consolidation. So, when the delay was longer in Experiments 2a and 2b, we observed a stronger implicit detection effect.

## Conclusion

Implicit detection was observed in all the main experimental conditions of this study. Specifically, the RTs of the change blindness trials were significantly larger than that of the baseline trials, regardless of whether there was a single or a combination of features change or the length of exposure durations and delays. When the exposure duration was prolonged, the difference between the RTs of the change blindness and baseline trials of the single feature change was significantly larger. In other words, the change of a single feature demonstrated a more obvious implicit detection effect at longer exposure durations. When the delay was prolonged, the difference between the RTs of the change blindness and baseline trials of the single and combination of features change became significantly larger. In short, with a longer delay, the implicit detection effect was stronger. Regardless of set size, the RTs of the blindness trials were significantly longer than those of the baseline trials. Set sizes had no significant impact on implicit detection because the capacity of pre-attention is infinite. To sum up, this study suggested in both single and combination of features change, and across all exposure durations and delays, that implicit detection occurs uniformly during the change blindness period. Furthermore, our results demonstrate that implicit detection occurs pre-attentively. These findings also provide a reference for subsequent studies in selecting appropriate stimulus types, exposure durations, delays, and set sizes.

## Data Availability Statement

The original contributions presented in the study are included in the article/supplementary materials, further inquiries can be directed to the corresponding author/s.

## Ethics Statement

The studies involving human participants were reviewed and approved by Ethical Committee of Guangxi University for Nationalities. Written informed consent to participate in this study was provided by the participants’ legal guardian/next of kin.

## Author Contributions

WX has done all the work, including but not limited to conceived the idea, designed the study, conducted the experiment, analyzed, and interpreted the data, and drafted the manuscript.

## Conflict of Interest

The author declares that the research was conducted in the absence of any commercial or financial relationships that could be construed as a potential conflict of interest.
